# *Medicago Mting1 Mting2* double knockout mutants are extremely dwarfed and never flower implicating essential MtING functions in growth and flowering

**DOI:** 10.1186/s12870-025-06432-x

**Published:** 2025-04-01

**Authors:** Matthew Mayo-Smith, Axel Poulet, Lulu Zhang, Yongyan Peng, David Goldstone, Joanna Putterill

**Affiliations:** 1 School of Biological Sciences, Faculty of Science, University of Auckland, Auckland Mail Centre, Private Bag 92019, Auckland, 1142 New Zealand; 2 Department of Molecular, Cellular and Developmental Biology, Faculty of Arts and Sciences, Yale University, New Haven, CT USA; 3 The New Zealand Institute for Plant and Food Research Limited (Plant & Food Research) Mt Albert, Auckland Mail Centre, Private Bag 92169, Auckland, 1142 New Zealand

**Keywords:** *INHIBITOR OF GROWTH*, MtING1, MtING2, *Medicago*, *Arabidopsis*, Flowering time, PHD finger, ING domain, NuA4 HAT

## Abstract

**Background:**

Optimal flowering time is critical to agricultural productivity. Despite this, flowering regulation in the Fabaceae (legume) family is not fully understood. For example, FLC and CO control Arabidopsis flowering, but do not regulate flowering in the temperate legume *Medicago*. Little is known about the genetic roles of the two plant *ING* genes. They encode proteins with conserved ING and PHD finger domains predicted to function as epigenetic readers. Previously, using CRISPR-Cas9 knock outs, we reported that *Medicago MtING2* promotes flowering and growth. However, surprisingly, *Mting2* PHD finger mutants flowered similarly to wild type. Additionally, *MtING1* did not regulate flowering because *Mting1* mutants flowered like wild type.

**Methods:**

To further dissect the combined genetic function of *MtING1* and *MtING2* and their PHD fingers, we cross-pollinated *Mting1* and *Mting2* single mutants to create two double mutants: The *Mting1-7 Mting2-2* double knockout mutant and the *Mting1-1 Mting2-11* double PHD finger mutant. Mutant phenotypes were assessed in floral-inductive conditions. We used fluorescence confocal microscopy and in vitro protein biophysical analysis to investigate the subcellular localization and oligomerization of the proteins. We carried out gene expression analysis by RNA-seq and RT-qPCR to determine how the two genes affect transcript accumulation to influence growth and flowering.

**Results:**

The *Mting* double knockout mutants displayed a striking, non-flowering, highly dwarfed phenotype indicating overlapping and complementary functions. Conversely *Mting* double PHD finger mutants showed only mild dwarfing and weak delays to flowering, indicating that the PHD fingers did not have a major impact on MtING function. MtING proteins localised to the nucleus, consistent with their predicted roles as histone readers, but did not interact in solution. Large changes to gene expression were seen in the *Mting2-2* single mutant and the double knockout mutant, with key flowering genes downregulated and predicted floral repressors elevated. Furthermore, the MtINGs promoted the expression of Medicago homologs of target genes of the Arabidopsis NuA4 HAT complex.

**Conclusions:**

Our findings demonstrate the key combined function the *MtING* genes play in regulation of global gene expression, flowering time and wider development and implicate an important role in epigenetic regulation via HAT complexes.

**Supplementary Information:**

The online version contains supplementary material available at 10.1186/s12870-025-06432-x.

## Background

The timing of flowering is a major factor in successful plant adaptation and crop productivity, including in the legume family which is the second most economically important plant group after the cereals [1]. Like winter annual *Arabidopsis thaliana* (L.) Heynh. (*Arabidopsis*), the temperate model legume *Medicago truncatula* Gaertn. (*Medicago*) is induced to flower by extended cold (vernalization, V) followed by warm long day (LD) conditions (VLD) [2]. However, strikingly in *Medicago* there is no CONSTANS that promotes flowering in LD, or FLOWERING LOCUS C to repress flowering like in *Arabidopsis* [3–6]. In addition, a predicted Polycomb complex PRC2 component MtVRN2 has a different function in flowering in *Medicago* to *Arabidopsis*, repressing the important *FLOWERING LOCUS T* (*FT*)-like gene *MtFTa1* prior to vernalization [7]. Despite this, other *Medicago* homologs appear to promote or repress flowering in a similar manner to *Arabidopsis*, such as the *Medicago* floral activators *MtFTa1*, *SUPPRESSOR OF CONSTANS 1* (*SOC1*) *MtSOC1a*, the photoreceptor MtPHYTOCHROME A which strongly promotes flowering in LD and *MtFE*, or repressors such as MtCYCLING DOF FACTORS [8–12]. Furthermore, the FT-FLOWERING LOCUS D (FD) complex is crucial for flowering in *Medicago* as double *Mtfta1 Mtfda* mutants never flower [13]. Gene edited *Mtsoc1a Mtsoc1b Mtsoc1c* triple mutants also do not transition to flowering, implicating a broader role for the duplicated *MtSOC1*-like genes in *Medicago* than *SOC1* in *Arabidopsis* [9, 14].

*INHIBITOR OF GROWTH* (*ING*) genes are found in most eukaryotes [15]. First identified as a tumour suppressor in humans [16], they have since been studied in other organisms such as yeast and are known to be involved in the regulation of gene transcription and development [17, 18]. The genes encode proteins with two conserved domains, a N-terminal ING domain and a C-terminal plant homeodomain (PHD) finger. The ING domain has been shown to facilitate protein–protein interactions, including to recruit activator histone acetyltransferase (HAT) or repressor histone deacetylase complexes to genes influencing their transcription [19–21]. For example, in yeast the YNG2 ING protein is a part of the NuA4 HAT [21–23]. The C-terminal PHD domain is a Cys4-His Cys3 zinc finger that binds to the chromatin mark H3K4me3 [24]*.*

Many plants, including *Medicago* and *Arabidopsis*, have two *ING* genes; *ING1* and *ING2* [25–27]. They encode proteins which are highly similar between plant species, with the *Arabidopsis* and *Medicago* proteins sharing 68% (ING1) to 80% (ING2) sequence identity, while only being ~ 30% identical between ING1 and ING2 within species [27]. The *Arabidopsis* ING proteins are present in the nucleus and can bind H3K4me3 peptides in vitro [25, 26, 28]. Binding studies and mass spectrometry indicate that *Arabidopsis* ING2 may be a part of a NuA4 complex, functioning as a scaffold in the Piccolo catalytic module [29–32], while AtING1 and OsING1 can interact with a plant-specific PAGA HAT complex [28]. *Arabidopsis Ating1* mutants show modest reduction in plant height and increased numbers of primary branches compared to wild type (WT), but no flowering phenotypes were reported [28]. No *Arabidopsis Ating2* mutant studies have been published yet, but recently we showed in *Medicago* that CRISPR-Cas9 gene-edited *Mting2* single knockout mutant plants were pale in colour and dwarfed [27]. They also had reduced *MtFTa1* expression and delayed flowering, especially in floral inductive VLD conditions compared to WT plants [27]. Analysis of a range of different gene-edited *Mting2* mutant plants indicated that an intact MtING2 ING domain was important for WT-like growth, development and flowering [27]. However, unexpectedly, we saw that mutations that affected the C-terminal PHD finger of MtING2, but left the ING domain intact, had only mild effects on plant growth and development [27]. Gene edited *Mting1* single knockout mutants also grew and flowered similarly to WT [27].

Here we make and characterise the *Mting1-7 Mting2-2* double knockout mutant. This has striking abnormal growth phenotypes and never flowers, indicating genetic redundancy as well as complementary functions. We identify differentially expressed genes by RNA-seq and RT-qPCR and compare these to *Arabidopsis* genes identified as potential NuA4 HAT targets [32]. We also investigate possible genetic redundancy of the PHD finger encoded by the *ING* genes by evaluating the *Mting1-1 Mting2-11* double PHD mutant.

## Methods

### Plant material and growth

Wild type *Medicago* R108_C3 (WT) [33] was used in this study. This was gifted from Richard Macknight (University of Otago, Dunedin, New Zealand). The *Mting1* and *Mting2* single mutants: *Mting1-1, Mting1-7, Mting2-2* and *Mting2-11,* were made by Matthew Mayo-Smith (Putterill laboratory, University of Auckland, Auckland, New Zealand) as reported in Jaudal et al*.* [27] (Table 1) and gifted to us for this project. The *Mting1 Mting2* double mutants were generated by crossing in this current study (Table 1) by authors Matthew Mayo-Smith and Lulu Zhang (Putterill laboratory, University of Auckland, Auckland, New Zealand).

**Table 1:** Description of *Mting1* and *Mting2* single and double mutants

line	predicted mutation effect	mutation description	predicted length (aa)	phenotypes
*Mting1-7*	predicted knockout	deletion causing a frame shift, encoding highly truncated protein	83	WT-like^a^
*Mting2-2*	predicted knockout	large deletion causing a frame shift, encoding highly truncated protein	44	small, late flowering, pale^a^
*Mting1-1*	PHD finger deletion	large deletion, removing encoded PHD finger	172	WT-like^a^
*Mting2-11*	PHD finger deletion	small indels, affecting encoded ING domain loop region and removing PHD finger	195	WT-like^a^
*Mting1-7 Mting2-2*	double knockout mutant		as above	tiny, no branches, never flowers
*Mting1-1 Mting2-11*	double PHD finger mutant		as above	mild developmental and flowering phenotypes

The predicted length of WT MtING1 is 247 amino acids (aa), and MtING2 is 263 aa

^a^according to Jaudal et al. [27]

To generate *Mting1-7 Mting2-2* double knockout mutant and the *Mting1-1 Mting2-11* double PHD finger mutant, the respective *Mting1* and *Mting2* mutant plants were manually cross-pollinated as previously described [34]. The combination of *Mting* alleles in the subsequent segregating F1 and F2 progeny was confirmed using allele specific genomic PCR (Table S1).

All *Medicago* plants used for this research were grown under VLD which is a floral-inductive condition. To sow plants, seeds were scarified using sandpaper (p600 grit), sterilised in a chlorine solution (Millipore, USA) for 10 min and germinated overnight, shaking in water at 15 °C in the dark. Germinated seeds were vernalized for 3 weeks at 4 °C on moist filter paper in a petri dish. Vernalized seedlings were planted directly into seed-raising mix (Daltons, NZ) in small 6-cell punnets placed on rockwool mats (Grodan, NE) sub irrigated with hydroponics [35] (without Na_2_O_3_Si). The plants were grown in a controlled greenhouse under white fluorescent light (~ 160–200 μmol m^−2^ s^−1^) in long days (16/8 h light/dark) at 22 °C.

*Nicotiana tabacum* L. (tobacco) was gifted to us by Nathan Deed (University of Auckland, Auckland, New Zealand) and used for transient expression of *35S:MtING-GFP*, *35S-GFP* and *35S:NLS-mCherry*. Seeds were sown at high density directly into seed raising mix and healthy seedlings were transplanted into 0.5 L pots with planting mix (Daltons) watered with hydroponics solution. The tobacco plants were grown under the same conditions as the *Medicago.*

### Phenotypic analysis

**Fig. 1:**
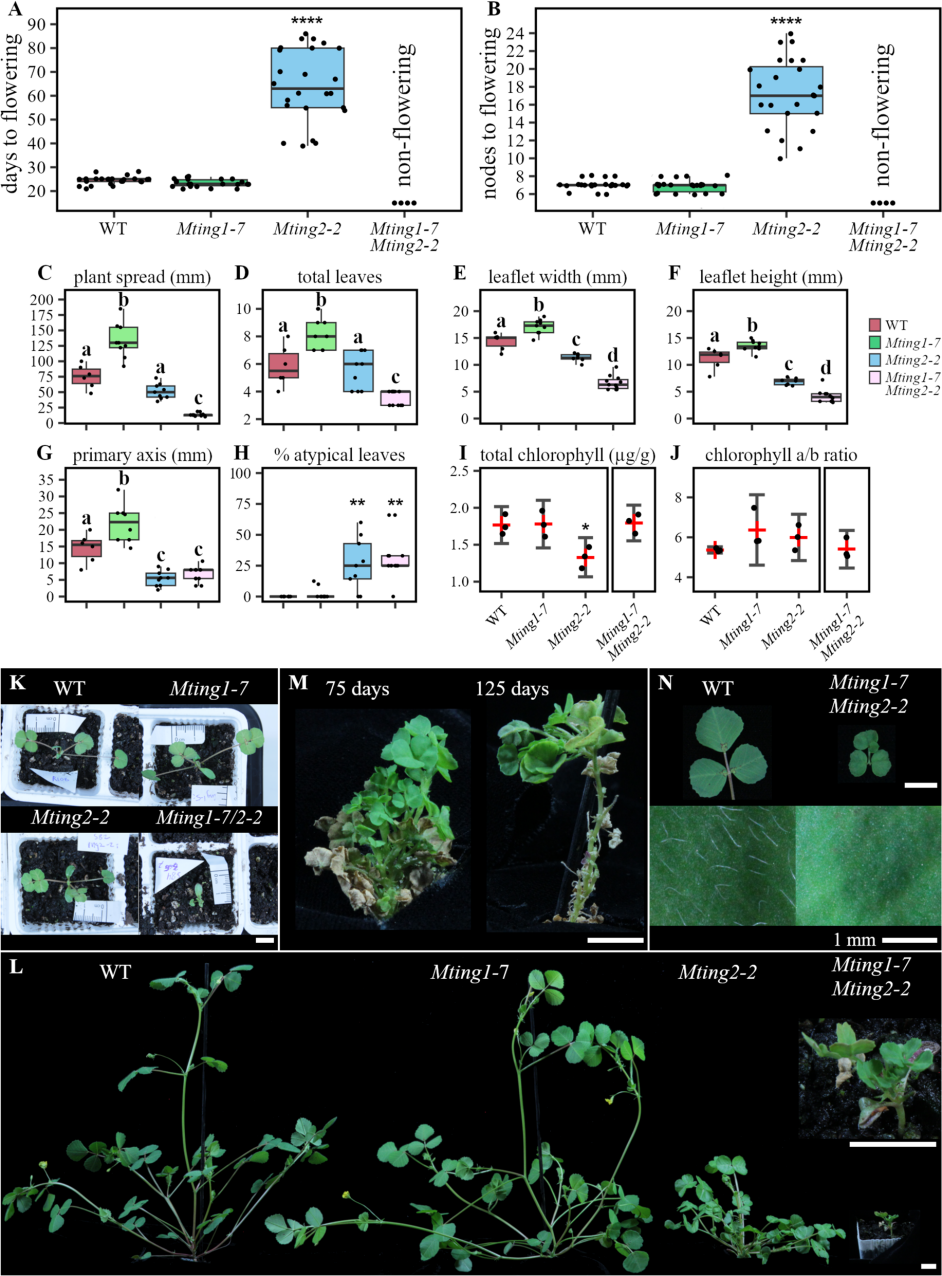
*Mting1-7 Mting2-2* double knockout mutants are very small and never flower. **A** Flowering time in days to flowering and (**B**) nodes to flowering in WT, *Mting1-7*, *Mting2-2*, and *Mting1-7 Mting2-2* in VLD. **C-H** Graphs showing six morphological traits; the distance from the monofoliate to furthest trifoliate (plant spread; **C**), total number of compound leaves (**D**), width (**E**), and height (**F**) of the largest terminal leaflet, primary axis height (**G**), and percentage of atypical (non-trifoliate) compound leaves on the plant (**H**) after 21 days in VLD. Data is shown as a boxplot with median and interquartile range. Statistical significance was determined using a Wilcoxon test (**A**,** B**, **H, I, J**; *p*-value with Bonferroni correction: *P ≤ 0.05, **P ≤ 0.01, ***P ≤ 0.001, ****P ≤ 0.0001) or one-way ANOVA and Tukey honest significant test (**C-G**). Groups with different letters are statistically different from each other. **I** The total chlorophyll concentration in µg per g of fresh weight and ratio of chlorophyll a to b (**J**) in the compound leaves of 33 day old WT, *Mting1-7* and *Mting2-2* or 233 day old *Mting1-7 Mting2-2* grown in VLD. Data is mean (red cross) and 95% confidence interval (whiskers) with replicates shown as dots. Statistical difference from WT was tested using a t-test assuming unequal variance. **K** Photographs from above of WT, *Mting1-7*, *Mting2-2*, and *Mting1-7 Mting2-2* after 14 days in VLD or from the side after 33 days in VLD (**L**). **M** The *Mting1-7 Mting2-2* mutant after 75 days and 125 days in VLD. **N** Comparison between trichomes on a terminal leaflet adaxial surface in WT and *Mting1-7 Mting2-2*. Scale bars are 1 cm unless labelled otherwise

**Fig. 2:**
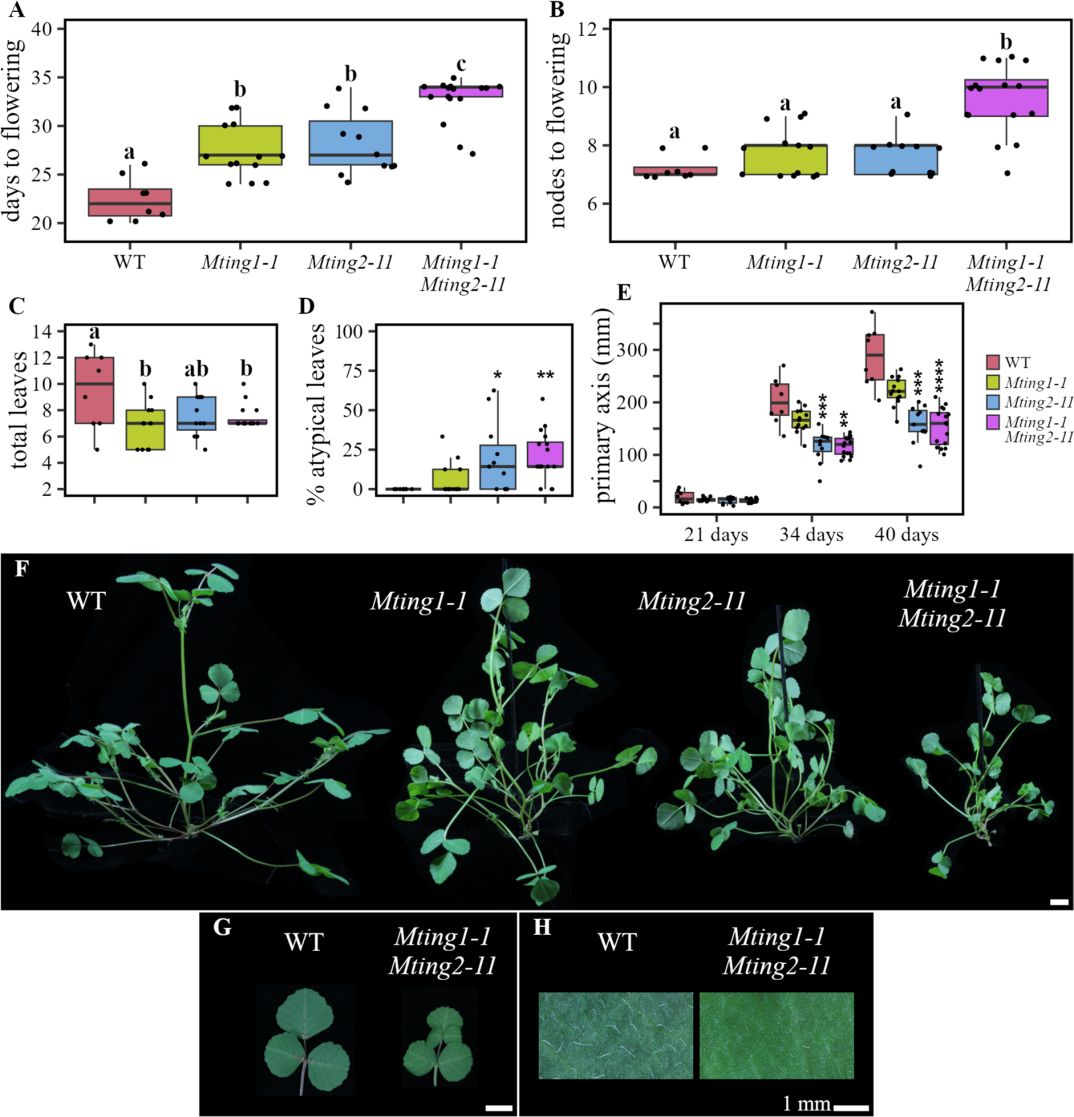
*Mting1-1 Mting2-11* double PHD mutants are smaller and later flowering than WT. **A** Flowering time in days to flowering and (**B**) nodes to flowering in WT, *Mting1-1*, *Mting2-11*, and *Mting1-1 Mting2-11* in VLD. **C** Graphs showing the total number of compound leaves and (**D**) percentage of atypical (non-trifoliate) compound leaves on the plant after 21 days in VLD. **E** Graph showing the height of the primary axis over time in VLD. Data is shown as a boxplot with median and interquartile range. Statistical significance was determined using a one-way ANOVA and Tukey honest significance test (**A-C**) (Groups with different letters are significantly different) or Wilcoxon test (**D**,** E**; *p*-value with Bonferroni correction: *P ≤ 0.05, **P ≤ 0.01, ***P ≤ 0.001, ****P ≤ 0.0001). **F** Photograph showing WT, *Mting1-1*, *Mting2-11* and *Mting1-1 Mting2-11* plants. **G** Typical compound leaf and (**H**) the adaxial leaf surface comparing trichomes on WT and *Mting1-1 Mting2-11* after 32 days in VLD. Scale bars are 1 cm unless labelled otherwise

The flowering time of plants was measured by the number of days after planting, and the number of nodes on the primary axis at the emergence of the first floral bud on the plant. For the flowering time data presented in Fig. 1, the sample size (n) ranged from 4 to 24 and in Fig. 2, 8 to 16. All other data is presented at a stated plant age where *n* = 6 to 11 for the data in Fig. 1, or 8 to 16 for Fig. 2. The plant spread was measured as the horizontal distance in millimetres from the monofoliate leaf tip to the leaf tip of the furthest branch. The total number of leaves was measured as the total number of fully expanded compound leaves plus the monofoliate leaf on the plant. The length of the primary axis was measured as the distance in millimetres from the base of the monofoliate node to the growth tip. The percentage of atypical leaves was measured as the number of non-trifoliate compound leaves (excluding the monofoliate) on the plant divided by the total leaf number. Trichome scoring was done by observing the presence/absence (Fig. 1) or visual density (Fig. 2) of leaf hairs on the adaxial and abaxial surfaces of fully expanded leaflets on > 3 week-old plants.

All phenotypic data is graphed as a boxplot with median and interquartile range. Statistical significance was tested with a one-way ANOVA and Tukey honest significance test (*p*-value ≤ 0.05) or where the data does not satisfy the assumptions of an ANOVA, a Wilcoxon test with Bonferroni correction testing for a difference to WT.

### Chlorophyll measurements

Chlorophyll content was measured according to Porra et al. [36] with modification. Chlorophyll was extracted from two leaves from a plant for each of the three biological replicates after 33 days in VLD for WT, *Mting1-7* and *Mting2-2*, or three replicates from different leaves on one *Mting1-7 Mting2-2* double mutant plant after 233 days. Two of the newest fully expanded compound leaves on the primary axis were harvested, weighed (approximately 100–150 mg) and frozen in liquid nitrogen. Chlorophyll was extracted from ground tissue using 2–5 ml of Tris–HCl pH 7 buffered 80% acetone. Samples were processed four at a time and diluted in additional buffered acetone so the absorbance at 663 nm was between 0.5 to 0.8. The absorbance at 646 nm and 663 nm (with correction at 750 nm) was measured using a Cary 4000 spectrophotometer (Agilent, USA). The total chlorophyll per gram of fresh weight and the chlorophyll a to b ratio was calculated according to Porra et al. [36]. The statistical significance from WT was tested using a t-test assuming unequal variance.

### Fluorescent protein localization assay

The full-length *MtING1* or *MtING2* coding sequence was cloned into a pHEX2 vector [37] modified for fusion with GFP at the C-terminal end of the encoded proteins under the cauliflower mosaic virus constitutive 35S promoter. A control *35S:GFP* was also cloned into pHEX2. A positive nuclear targeted control *35S:NLS:mCherry* was also cloned using Golden Gate assembly [38]. The vectors were transformed independently into *Agrobacterium tumefaciens* strain GV3101 and cultured. *A. tumefaciens* containing the respective *GFP* and *mCherry* vector were mixed in equal parts and resuspended into infection medium (50 mM MES pH 5.6, 2 mM Na_2_HPO_4_, 28 mM glucose, 200 µM acetosyringone), and injected into the two newest leaves of 5–7 leaf stage tobacco plants. Small circular discs were sampled from the infiltrated leaves after three or five days. Transformations were repeated in two independent experiments with three independently transformed plants per experiment.

Confocal laser scanning microscopy images were captured on an Eclipse Ti-E microscope with a 40 × S Plan Fluor ELWD objective lens (Nikon, Japan), CSU-X1 confocal scanner unit (Yokogawa, Japan), ALC-500 laser unit and Zyla sCMOS camera (Andor, UK). GFP and mCherry were excited using a laser light wavelength of 488 nm and 561 nm, respectively, and images captured with a 3035B and TX Red 4040B filter (Semrock, USA). For each sample, greater than 100 cells from each leaf disc were observed. Single slice images were processed using NIS-Elements imaging software (Nikon, v4.06.12).

Potential nuclear localization signal (NLS) sites for the MtING proteins were identified using NLStradamus [39]. The full-length sequences of human ING4, MtING1 and MtING2 were queried using a 4-state HHM static model with 0.6 threshold on the NLStradamus webserver [39].

### SEC–MALLS

Size exclusion chromatography (SEC) with multi angle laser light scattering (SEC–MALLS) was used to determine the weight-average molecular weight (MW) and oligomeric state of recombinant MtING ING domain protein in solution. The coding sequence of the ING domain of MtING1 and MtING2, named MtING1_ING_ (M1-E118) and MtING2_ING_ (M1-E133), was cloned from WT into a pET-49b( +) vector for N-terminal fusion with either THIOREDOXIN (TRX:MtING1_ING_) or MALTOSE BINDING PROTEIN (MBP:MtING2_ING_). The induction of recombinant protein expression was the same as previously described [27]. Protein was purified from cell lysate using either nickel immobilised metal affinity chromatography (TRX:MtING1_ING_) or amylose affinity chromatography (MBP:MtING2_ING_) followed by SEC, and then concentrated to 4.25 mg/mL using an Amicon 3 K centrifugal concentrator (Merck, De).

Purified samples (100 µL) were analysed using SEC–MALLS at 25 °C with a Dionex UltiMate 3000 HPLC pump (Thermo Fisher, USA), SLD7000 7-angle MALL detector (PSS, De), RI-101 differential refractive index detector (Shodex, De) and Superdex 200 Increase 10/300 GL 24 mL column (General Electric, USA) in MALLS buffer (10 mM Tris–HCl pH 8, 150 mM NaCl, 0.1 mM TCEP, 3 mM azide). The MW was determined using PSS winGPC Unichrom software.

### Plant tissue harvesting and RNA extraction

For RNA-seq and real time reverse transcription quantitative PCR (RT-qPCR), leaf and shoot apex tissue was harvested from plants grown under VLD conditions, 4 h after dawn on day 14 (WT, *Mting1-7*, *Mting1-1*, *Mting2-11* and *Mting1-1 Mting2-11*), day 15 (*Mting2-2*) and day 21 (*Mting1-7 Mting2-2*) when the plants had 2–3 fully expanded compound leaves. Three biological replicates were harvested per tissue type per genotype, each consisting of 1 to 2 fully expanded compound leaves from the same plant, or 3 to 4 primary apices from different plants. Harvested tissue was snap-frozen in liquid nitrogen and homogenised by metal beads in a Geno/Grinder® 2010 (New Jersey, USA). Total RNA was extracted using the RNeasy Plant Mini Kit (Qiagen, De) according to the user manual. RNA quantity and quality were checked by a NanoPhotometer® N60 (Implen, De) and a Bioanalyzer 2100 (Agilent Technologies, USA).

### RNA-seq and analysis

The RNA-seq was carried out by Novogene (Hong Kong) with library preparation and sequencing as previously described [14]. Directional strand specific mRNA libraries (poly A enrichment) were prepared and sequenced on the Illumina NovaSeq platform NovaSeq6000, 150 bp paired-end (Novogene, Hong Kong). The FASTQ file read quality was evaluated, and Fastp (v0.21) was used for trimming [40]. Reads with a quality below a PHRED score of 20 were trimmed from the 3’end and reads < 36 bp in length were excluded. The remaining reads were mapped against the Mt4.0v2 transcriptome [41, 42] using Salmon (v0.8.2). DESeq2 (v1.24.0) [43] was used for normalization and differential expression analyses. Differentially expressed transcripts were filtered using a cut-off adjusted *p*-value ≤ 0.05 and log2 fold-change ≥ 1 or ≤ − 1. To identify candidate direct target genes of NuA4, we used Blastx [44] against *Arabidopsis* NuA4 bound and regulated genes [32]. All the genes with an e-value < e − 100 were selected. Genes that were not expressed or only weakly expressed were removed, which gave a list of 289 transcripts (Table S5).

### Gene expression analysis by RT-qPCR

The WT and *Mting1-7 Mting2-*2 RNA samples were treated with DNase (TURBO DNA-*free*™ Kit, Invitrogen, USA) according to manufacturer's instructions. Template cDNA was synthesised with SuperScript™ IV Reverse Transcriptase (Invitrogen) as previously described [8, 10]. Gene expression analysis was performed based on the comparative CT method [45], with modifications [14, 46]. ΔCT values were obtained by normalising genes of interest to the reference gene, *PROTEIN PHOSPHATASE 2A* (*PP2A*, Medtr6g084690). Relative gene expression was calculated using the formula 2^−ΔCT^. Statistical significance between WT and *Mting1-7 Mting2-2* was calculated using the t-test, assuming unequal variance (*p*-value ≤ 0.05). RT-qPCR primers are listed in Table S1.

## Results

### *Mting1-7Mting2-2* double knockout mutants have severe growth and developmental defects and never flower

Previously, we showed that *Mting2* single knockout mutations negatively impacted growth and delayed flowering in *Medicago*, but *Mting1* single mutants were like WT [27]. To assess the physiological consequences of strong mutations in both *MtING* genes, we generated a double knockout mutant by crossing *Mting1* and *Mting2* single knockout mutants. The *Mting1-7* single knockout mutant carried a deletion causing a frameshift in the encoded protein. This led to an altered amino acid (aa) sequence from aa 21 onwards and a truncated protein of 83 aa, compared to 247 aa WT MtING1 (Table 1). The small, pale green and late flowering *Mting2-2* single knockout mutant was predicted to encode a truncated protein with a deletion of aa 16 to 39 and a final protein of 44 aa, compared to WT MtING2 of 263 aa (Table 1). After crossing, we grew F1 plants and then identified homozygous *Mting1-7 Mting2-2* double mutant F2 progeny by PCR genotyping (Table S1).

The double mutants had multiple highly abnormal phenotypes, compared to the single mutant parents and WT (Fig. 1). As expected, in the parental lines, *Mting1-7* flowered like WT, while *Mting2-2* flowered late in floral inductive VLD conditions. Conversely, the *Mting1-7 Mting2-2* double knockout mutant never produced a floral bud (Fig. 1A/B). The double mutants continued to grow very slowly but still had not flowered after six months after which they started to die. The double mutants were severely dwarfed even when compared to the small *Mting2-2* mutant (Fig. 1K/L). The *Mting1-7 Mting2-2* plants also had significantly reduced plant diameter (plant spread), smaller leaflets and fewer compound leaves compared to *Mting2-2* (Fig. 1C-F). The double mutants could not maintain outgrowing branches, because they withered as the plants grew (Fig. 1M). Like the *Mting2-2* single mutant parent [27], they had an increase in the percentage of atypical compound leaves and no leaf trichomes (Fig. 1H/N). Therefore, this double mutant analysis implicates both *MtING* genes as essential for normal development in *Medicago*, with redundant and complementary roles in the regulation of flowering, growth and plant architecture.

*Mting1-7* single mutants had a slight increase in plant size when compared to WT in five of the measured morphological traits (Fig. 1C-G) at 21 days in VLD. This indicates that *Mting1-7* in an otherwise WT background appears to have a weakly promotive effect on *Medicago* plant size, unlike *Mting2-2,* or *Ating1* mutants which are dwarfed compared to WT [28]. Previously, we reported that *Mting2* mutants had a pale colour [27]. To determine if this was the result of changes in chlorophyll levels, we measured the chlorophyll content in the leaves of the mutants compared to WT. *Mting2-2* mutants had a reduced total chlorophyll amount compared to WT and *Mting1-7*, but there was no change in the ratio of chlorophyll a to chlorophyll b for any plants indicating that *Mting2* affects the production of both chlorophylls equally (Fig. 1I/J). In contrast, the double knockout mutant displayed similar chlorophyll levels to WT (Fig. 1I/J).

### *Mting1-1Mting2-11* double PHD mutants show only mild growth and flowering phenotypes but a strong reduction in leaf trichomes

Previously, our analysis of a range of gene-edited *Mting2* single mutants indicated that the ING domain of MtING2 was important for normal growth and flowering in *Medicago* [27]. But, unexpectedly, *Mting2* mutants with changes to or loss of the PHD finger domain grew similarly to WT, indicating that the MtING2 PHD finger domain was not crucial in an otherwise WT background [27].

As demonstrated above, *Mting1-7 Mting2-2* double knockout mutants had a much stronger abnormal growth and development phenotypes than the parental lines, indicating that *MtING1* and *MtING2* had overlapping and complementary functions. Thus, we hypothesised that *Mting* double PHD finger mutants may also exhibit enhanced mutant phenotypes compared to the single PHD finger mutants. To test this, we crossed *Mting2-11* plants with mutations predicted to only affect the PHD coding sequence and the non-essential loop region of the ING domain and which grew similarly to WT, to *Mting1-1* plants with mutations predicted to remove large portions of the PHD finger, but not affecting the ING domain and which also grew like WT (Table 1) [27]. After crossing, we selected the *Mting1-1 Mting2-11* double PHD finger mutants in the F2 generation by PCR genotyping (Table S1). Phenotyping of the *Mting1-1 Mting2-11* double PHD mutants (Fig. 2) showed that they flowered slightly later in both days and nodes to flowering than WT and the parents (Fig. 2A/B). The parental lines showed a mild delay to flowering in days, but not in nodes compared to WT. The *Mting1-1 Mting2-11* double PHD mutants still flowered much earlier than the *Mting2-2* knockout plants (Fig. 1A/B). This suggested that the ING PHD fingers made a mild combined contribution to the promotion of flowering but had a much weaker effect than the intact ING proteins. In addition, the *Mting1-1 Mting2-11* double PHD mutant plants and their compound leaves were more compact than WT and the single mutant parental lines (Fig. 2F/G), indicating again a mild contribution of both MtING PHD fingers on plant growth. Interestingly, the *Mting1-1 Mting2-11* double PHD mutants also displayed greatly reduced visual leaf trichome density compared to WT (Fig. 2H). The reduction in trichomes was like the *Mting1-7 Mting2-2* double knockout mutants (Fig. 1N). This implicates both MtING PHD fingers as vital for normal leaf trichome development. In other phenotypes, the *Mting1-1 Mting2-11* double PHD mutant and the *Mting2-11* parent had a similarly slightly shorter primary axis and a higher percentage of atypical compound leaves, compared to WT and *Mting1-1* (Fig. 2D/E). This suggested that loss of the MtING2 PHD finger alone was sufficient for this mild dwarfing and change in leaf patterning. Compared to WT, total leaf number was slightly reduced to a similar level in *Mting1-1* and the *Mting1-1 Mting2-11* double PHD mutant (Fig. 2C).

In summary, *Mting1-1 Mting2-11* double PHD mutants had weakly abnormal growth and delayed flowering phenotypes and strong loss of leaf trichomes.

### MtING proteins localize to the nucleus in tobacco cells but do not dimerizein vitro

Human ING proteins contain a nuclear localization signal (NLS) in the disordered linker between the PHD finger and N-terminal ING domain and are nuclear localized [47]. Furthermore, some human ING proteins contain an additional nucleolar localization signal (NoLS) within the NLS or downstream of the PHD finger [48–50]. Previously, it was shown that *Arabidopsis* AtING1 and AtING2 GFP-tagged proteins located to the nucleus in transient expression in protoplasts, although the NLS and NoLS identified in human INGs were not found in these ING proteins [25, 26].

**Fig. 3:**
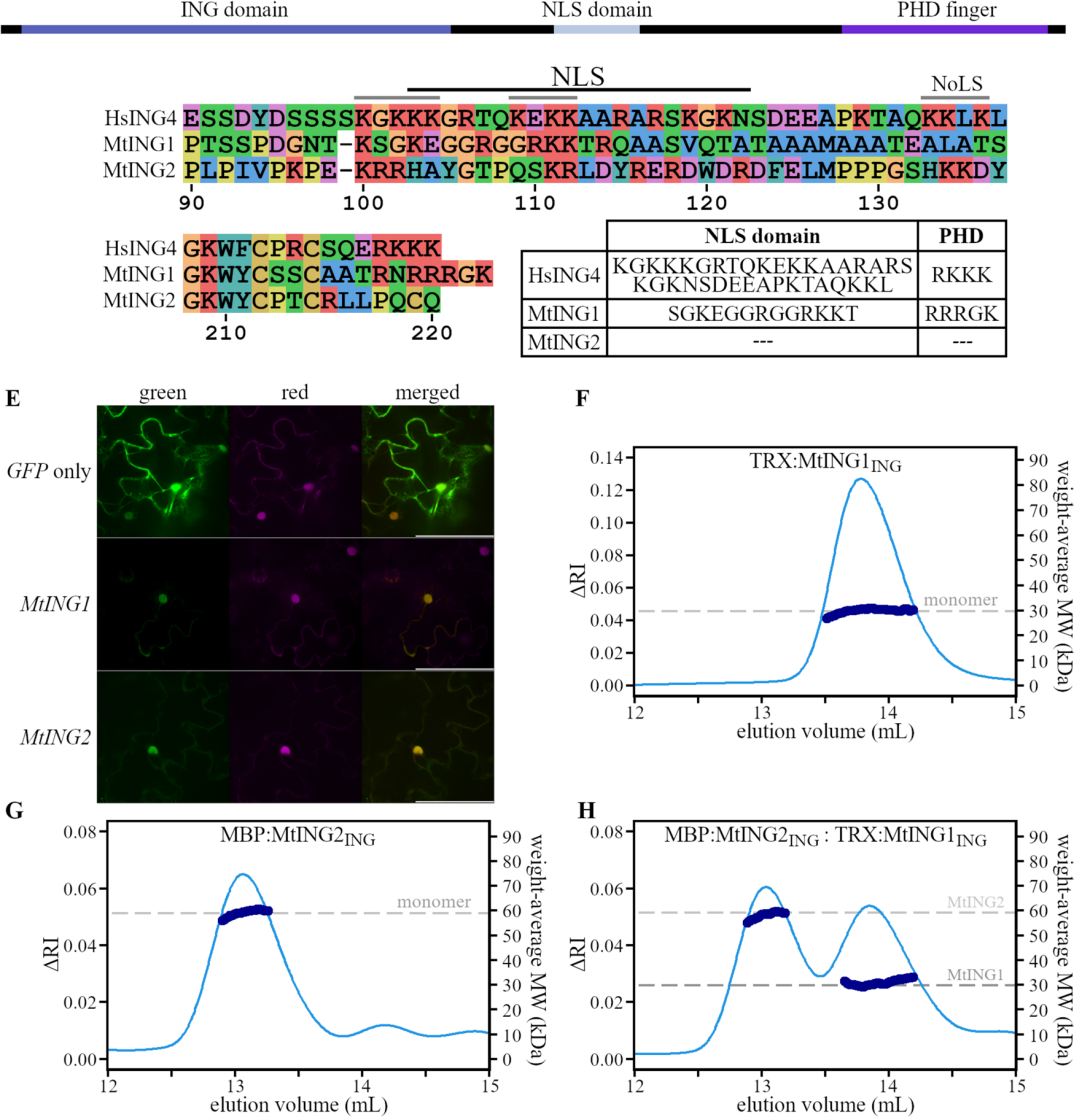
MtING proteins are nuclear localized but do not interact in vitro to form dimers. **A** Schematic diagram showing the location of the nuclear localization signal (NLS) domain in HsING4. **B** Protein sequence alignment of the human HsING4 NLS domain and the homologous *Medicago* ING regions and (**C**) end of the PHD finger. The HsING4 NLS (black line) and nucleolar localization sequence (NoLS, grey lines) identified by Tsai et al. [49] are shown. The numbering is based on the relative position within ING4. **D** A table showing potential predicted NLS peptides using NLStradamus [39]. **E** Confocal microscopy images from tobacco explants co-transformed with *NLS:mCherry* (red channel, magenta) and either *GFP*, *MtING1:GFP* or *MtING2:GFP* (green channel, green) overexpression constructs. Photographs are representative of two separate experiments each with three independent transformations. Scale is 100 µM. **F** SEC–MALLS was used to analyse if tagged MtING proteins dimerised in vitro. Graph showing the elution profile for TRX-tagged MtING1 ING domain (TRX:MtING1_ING_) and (**G**) MBP-tagged MtING2 ING domain (MBP:MtING2_ING_) recombinant protein at a concentration of ~ 4.25 mg/ml (289 µM or 62.8 µM for ING1 or ING2, respectively) or (**H**) 1:1 equimolar (51.4 µM) mixture of MBP:ING2_ING_ and TRX:MtING1_ING_. The protein elution profile (light blue) is measured by the change in refractive index (∆RI) and the calculated weight-average molecular weight (dark blue) is in kilodaltons. The expected molecular weight of monomeric tagged MtING_ING_ protein is indicated by a dashed grey line

Here, we first compared the known NLS from human ING4 to the homologous regions of MtING1 and MtING2 and used in silico analysis to predict NLS in the *Medicago* proteins (Fig. 3A-D). Both Medicago INGs contain an enrichment of basic R and K residues in the NLS domain region which is similar to that of the human ING4 NLS (Fig. 3B) [47, 49]. Furthermore, MtING1, but not MtING2, has a RK rich sequence down downstream of the PHD finger, like the second NLS of ING4 (Fig. 3C) [50]. This is supported by the in silico prediction which identified both sites as potential NLS for MtING1. No sites were predicted for MtING2 (Fig. 3D). Therefore, to empirically test if MtING1 and MtING2 are nuclear localised, we generated overexpression constructs designed to express MtING1 and MtING2 translational fusions with C-terminal GFP and used Agroinfiltration for transient expression in tobacco leaves. A *GFP* construct was infiltrated as a positive GFP control, while a *NLS-mCherry* construct was co-transformed with the *GFP* constructs and provided a positive nuclear control. Confocal microscopy (Fig. 3E) indicated that both MtING1 and MtING2 fusion proteins are nuclear localized, like the control NLS-mCherry. In contrast the control GFP was located to both nucleus and cytoplasm.

Studies of three of the human ING proteins most closely related to the plant INGs have shown that they form homodimers and heterodimers through the N-terminal ING domain in solution [20, 51, 52]. Thus, it is possible that plant ING proteins are also physically interacting through the ING domain. If the plant ING proteins do form homo or hetero dimers *in planta* this could help to explain why mutation of both *MtING* genes is required for some of the double mutant phenotypes. To test this, the ING domain coding sequence from *MtING1* and *MtING2* was cloned and fused to a recombinant N-terminal tag sequence (TRX or MBP) for expression in *E. coli*. Purified MtING-ING domain protein (MtING_ING_) was then tested for oligomerization in vitro using SEC–MALLS analysis. When analysed alone, recombinant TRX:MtING1_ING_ or MBP:MtING2_ING_ eluted with a predicted weight-average molecular weight (MW) consistent with monomeric proteins (Fig. 3F/G). Furthermore, an equimolar mixture of both proteins eluted as two separate peaks with each peak having a MW consistent for two independent monomeric proteins (Fig. 3H). Thus, the ING domain of the MtING proteins does not dimerise in solution either as homo- or hetero-dimers.

In summary, while both ING proteins are in the plant cell nucleus, the SEC–MALLS analysis indicated that the MtING proteins did not interact to form either homodimers or heterodimers in solution in vitro.

### Gene expression analyses in the *Mting1-7Mting2-2* double knockout mutant is consistent with its poor growth and non-flowering phenotypes

**Fig. 4:**
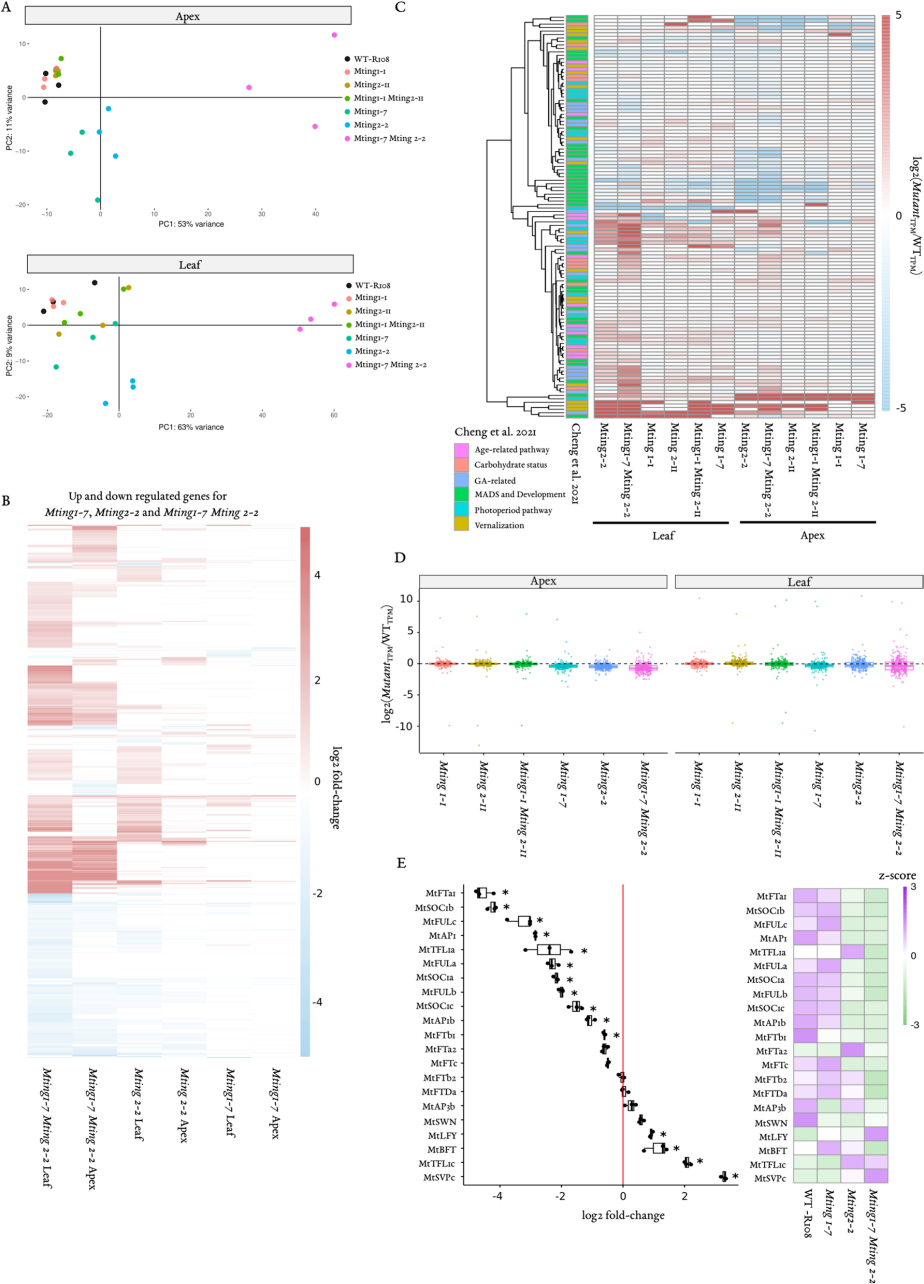
Gene expression in *Mting1-7 Mting2-2* is consistent with non-flowering and has similarities to *Atepl1*. **A** PCA plots for apex and leaf tissue RNA-seq data from the single parental *Mting* mutant lines, double *Mting* mutants and WT. Three biological replicates were harvested for each genotype. **B** A heat map of the log2 fold change obtain with the deseq2 analysis of all up and down differentially expressed genes in *Mting1-7*, *Mting2-2* and *Mting1-7 Mting2-2* relative to WT and (**C**) 93 selected candidate genes extracted by Cheng et al*.* [13] in all mutants relative to WT. The data is represented as the log2 of *Mutant*_TPM_/WT_TPM_. **D** Boxplot of the log2(*Mutant*_TPM_/WT_TPM_) in all *Mting* mutants for *Medicago* homologs of *Arabidopsis* genes targeted by NuA4. The *Arabidopsis* genes were previously identified as targets of NuA4 by RNA-seq and ChIP-seq in the *Atepl1* NuA4 mutant and WT [32]. **E** RT-qPCR analysis (left panel) and RNA-seq (right panel) of 21 candidate flowering genes in *Mting* mutant apex tissue. The left panel shows the boxplot of log2 fold-change relative to WT. Asterisks indicate a significant difference in gene expression between WT and mutant by t-test, assuming unequal variance (*P* ≤ 0.05). The right panel is expression shown as Z scores extracted from the TPM values of the RNA-seq

To further investigate the molecular basis of the strongly abnormal phenotypes of the *Mting1-7 Mting2-2* double knockout mutant, we analysed leaf and shoot apex gene expression in the double mutant, other *Mting* mutants and WT in VLD by RNA-seq and RT-qPCR (Tables S1-S5, Fig. 4). As expected from the strong mutant phenotypes, gene expression in the *Mting1-7 Mting2-2* double knockout mutant differed the most from WT out of all the mutant genotypes. It was strongly separated from all other genotypes in the Principle Component Analysis (PCA) (Fig. 4A) with thousands of differentially expressed genes (DEGs) compared to WT; 5189 and 3017 upregulated genes and 3064 and 1427 down regulated genes, in leaves and shoot apices respectively (Table S2, Fig. 4B). The *Mting2-2* single knockout mutant also separated distinctly from WT in the PCA analysis and had the next highest number of DEGs; 2106 and 660 upregulated and 758 and 326 genes down regulated in leaves and shoot apices respectively compared to WT (Table S2, Fig. 4A/B). This is consistent with the abnormal growth and developmental phenotypes of *Mting2-2*. In contrast, the *Mting1-7* single knockout mutant clustered separately from WT in apex, but not in the leaves, and had fewer DEGs; 504 and 159 upregulated and 250 and 143 down regulated genes in leaves and shoot apices respectively compared to WT (Table S2, Fig. 4B). The remaining *Mting* single and double PHD mutants, clustered with WT in both the leaf and shoot apex samples in the PCA. This included the *Mting1-1 Mting2-11* double PHD finger mutant with 584 and 140 up regulated genes and 124 and 41 downregulated genes in the leaf and shoot apex respectively. The most strongly down-regulated gene (Table S2, S4) in *Mting2* single and double mutants relative to WT, in shoot apices and/or leaves, was a candidate HAT gene Medtr5g017020. The predicted encoded protein is of the HAT-KAT11 superclass, in the p300/CBP HAT family (Table S2, S4). Interestingly, a second, HAT related gene, Medtr5g085310 (Table S2), was strongly down regulated in all *Mting2* single and double mutants in both tissues. This gene encodes a protein of the HAT-KAT11 superclass in the Zf-TA2 family. Amongst the most strongly up-regulated genes, we noted that in most *Mting2* mutants in leaves, these included stress-related genes encoding heat shock protein 70 (Table S2, S4) and a MYB/SANT domain gene (Table S2).

Given that the *Mting1-7 Mting2-2* double knock out mutant did not produce flowers, and two other *Mting* mutants had delayed flowering, we then examined the expression of candidate *Medicago* flowering regulators [27] in these mutants in RNA-seq and by RT-qPCR (Table S2, S4, Fig. 4C/E). In the *Mting1-7 Mting2-2* double knockout mutant many MADS genes involved in flowering were expressed at lower levels in the RNA-seq in shoot apices relative to WT. These included the inflorescence meristem identity genes *MtAP1* and *MtAP1b*, the I2 meristem identity gene *MtFULc* and paralogs *MtFULa-b* as well as *MtSEP1,4* and *MtSOC1a-c.* Conversely, candidate floral repressors such as *SVP-*like genes and *MtTEM1* and *MtTEM2* were elevated in the mutant. Similarly*,* further analysis of expression of 21 candidate flowering genes by RT-qPCR (Fig. 4E) revealed that 15 of them were significantly differentially expressed (11 down and 4 up) in the *Mting1-7 Mting2-2* double knockout mutant relative to WT. The down regulated genes included the MADS genes above, as well as the potent floral activator *MtFTa1* and paralog *MtFTb2,* and *MtTFL1a* which promotes primary inflorescence identity. Up regulated genes included the candidate floral repressors *MtBFT, MtTFL1c* and *MtSVPc.* RNA-seq of these genes generally followed the same pattern (Fig. 4E). The *Mting2-2* single knockout mutant showed strongly delayed flowering. It had reduced expression of *MtFTa1* and the MADS floral activators and elevated expression of candidate repressors in the RNA-seq as previously reported [27]. The *Mting1-1 Mting2-11* double PHD mutant had a mild delay to flowering. Amongst the MADs box genes, this correlated with down regulation of the important flowering regulator *MtAP1* and *MtSEP4* compared to WT. On the other hand, the *Mting1-7* single knockout mutant flowered similarly to WT and had a similar expression of candidate flowering regulators compared to WT.

Since the strong mutant phenotypes and abnormal gene expression of the *Mting* knockout mutants compared to WT indicated major disruption to regular biological processes, we then carried out a Gene Ontology enrichment analysis for the genes up and down regulated in the *Mting1-7 Mting2-2* double knockout mutant and other *Mting* mutants compared to WT (Table S3). We saw that the differentially expressed genes in *Mting1-7 Mting2-2* were enriched in many biological processes. In the upregulated genes these included pathways related to plant signalling and stress such as defence and biotic stimulus responses, consistent with the upregulation of the heat shock protein 70. Down regulated enriched processes included photosynthesis, chloroplast-related and metabolism, which was consistent with the very poor growth of the mutant. In the *Mting2-2* single knockout mutant, up-regulated enriched pathways in the leaf included core processes such as rRNA processing, while in the shoot apex, upregulated genes were enriched for core processes such as transcription by RNA Polymerase III and in plant responses to biotic stimuli and defence chemical metabolism, like the *Mting1-7 Mting2-2* double knock out mutant. Downregulated genes in *Mting2-2* in both tissues had similarities to the *Mting1-7 Mting2-2* double knockout mutant with enrichment in pathways related to photosynthesis and metabolism. The *Mting1-7* mutant had a different profile with upregulated genes in the apex showing enrichment in processes including cellular amide metabolic processes, while in leaf there was enrichment in lipid metabolic processes. There was enrichment in amine biosynthetic processes in apex down regulated genes but no enrichment amongst the down regulated genes in the leaf.

Although there have been no described *Arabidopsis Ating2* mutants, AtING2 has been shown to interact with components of the NuA4 HAT complex [29–32]. Furthermore, examination of the NuA4 scaffold protein Enhancer of Polycomb-Like 1 (AtEPL1) double a and b mutant (*Atepl1*) showed that the plants were small, pale and had a downregulation in genes related to the chloroplast [31, 32, 53]. This is quite similar to the phenotype and differentially expressed genes we saw in the *Mting2-2* single knockout and *Mting1-7 Mting2-2* double knockout mutants (Table S3, Fig. 1). Therefore, we examined if *Medicago* homologs of a list of gene targets of NuA4 in *Arabidopsis* were differentially expressed in the *Mting1-7 Mting2-2* double knockout mutant and respective single mutants. The *Arabidopsis* NuA4 targeted genes were defined previously by a loss of H4K5 acetylation and downregulation in the mutant compared to WT, as determined by ChIP-seq and RNA-seq [32]*.*

Interestingly, in the *Mting1-7 Mting2-2* double knockout mutant and respective single mutants, there was a marked down regulation of the selected genes in the apex tissue, with > 87% of the genes below a log2(*Mutant*_TPM_ / WT_TPM_) of 0 (Table S5, Fig. 4D). The magnitude of the movement was also greater in the double mutant, with a median value of −0.72 compared to −0.38 and −0.44 in the *Mting1-7* and *Mting2-2* single mutants, respectively. This is opposite to the general pattern of all apex DEGs, which were majority upregulated in *Mting2-2* and *Mting1-7 Mting2-2* or neutral in *Mting1-7* (Table S3, Fig. 4B). There was a similar but weaker pattern in the leaves, with log2(*Mutant*_TPM_/WT_TPM_) median values of −0.25, −0.20 and −0.33 in the *Mting1-7*, *Mting2-2* and *Mting1-7 Mting2-2* mutants respectively. Overall, this indicates that the MtINGs promote the expression of *Medicago* homologs of the NuA4 *Arabidopsis* target genes in WT. Conversely, none of the PHD finger mutants skewed in either direction (Table S5, Fig. 4D), suggesting that the PHD finger does not contribute to the regulation of this subset of genes.

## Discussion

The *ING* gene family is an important group of eukaryotic genes that are involved in the epigenetic regulation of a broad range of cellular processes such as gene regulation, apoptosis, senescence, cell cycle and rRNA synthesis [18, 50]. Despite this, relatively little is known about the genetic function of the two plant *ING* genes, *ING1* and *ING2*, especially in regard to the regulation of flowering time which is critical for crop productivity.

### Analysis of the *Mting1-7Mting2-2* double knockout mutant indicates combined essential roles of the two MtING genes in flowering

To assess the combined role of the *Medicago ING* genes *MtING1* and *MtING2* in flowering time and growth we generated *Mting* double knockout mutants. The late flowering, short stature *Mting2-2* mutant was crossed to the WT-like *Mting1-7* mutant. The resulting *Mting1-7 Mting2-2* double knockout mutants were highly dwarfed, did not maintain outgrowing branches and never flowered. Previous genetic studies of other key *Medicago* flowering genes have shown that *Mtfta1 Mtfda* double mutants and *Mtsoc1a-c* triple mutants are also non-flowering [13, 14]. Both mutants produced one lateral structure at the leaf axil indicative of remaining vegetative [13, 14]. Because the branches of *Mting1-7 Mting2-2* mutants withered shortly after initiating, we could not determine the lateral structures in the axil. However, gene expression analysis in the shoot apex showed that key activators upstream of the floral transition including *MtFTa1* and all three *MtSOC1* genes, and markers of *Medicago* compound inflorescence meristem identity including *MtAP1, MtTFL1a* and *MtFULc* genes were significantly downregulated compared to WT (Fig. 4). Furthermore, the putative flowering repressors *MtSVPc*, *MtTFL1c* and *MtBFT* were upregulated. In addition, *Mting1-7 Mting2-2* showed > 2 times as many differentially expressed genes amongst candidate flowering gene lists than other *Mting* mutants, including the late flowering *Mting2-2* single mutant. The combined misexpression of some of these genes, together with the greatly reduced *MtTFL1a* expression and elevated *MtSVPc* expression compared to the *Mting2-2* single mutant, likely contributed to the double knock out mutant remaining vegetative. In summary, the *Mting1-7 Mting2-2* double knockout mutant is highly dwarfed and is unable to transition to flowering. This is overall strikingly different from the single mutant parents, *Mting2-2* with delayed flowering and small stature and *Mting1-7* which appears similar to WT, indicating combined essential roles of the two *MtING* genes in flowering.

### Combined MtING gene effects on growth and architecture

Beyond flowering time, the strong and diverse phenotypes of the *Mting1-7 Mting2-2* double knockout mutant including great reduction in plant height, changes to compound leaf morphology, loss of branching and large global changes to gene expression compared to WT and the single mutant parents, suggests that the two *MtING* genes have a combined strong pleiotropic effect on gene expression and plant development. Biochemical analyses in *Arabidopsis* suggested that AtING2 is a scaffold component of the NuA4 HAT complex with roles in promoting chloroplast development and photosynthesis [31, 32, 36, 53]. Similarly, photosynthesis and chloroplast-related processes were enriched amongst the down regulated genes in the *Mting2-2* single mutant and in the *Mting1-7 Mting2-2* double knockout mutant (Table S3). There was an overall reduction in the level of expression of *Medicago* homologs of *Arabidopsis* NuA4 target genes particularly in the shoot apex in the *Mting1-7*, *Mting2-2* and *Mting1-7 Mting2-2* double mutant compared to WT, with a greater magnitude of reduction in the double mutant (Fig. 4D). Interestingly, stress response processes were enriched in the upregulated genes in *Mting2-2* and *Mting1-7 Mting2-2.* This is also seen for NuA4 mutants such as *Atepl1* [32]*.* These results are overall consistent with the idea that MtING1 and MtING2 may regulate gene transcription through histone modification such as through NuA4 action. However, *Arabidopsis* AtING2 can also bind to Histone Deacetylase Complex 1 (HDC1) [54] a possible component of plant histone deacetylase machinery [55], while AtING1 can bind to SUBUNIT OF THE PAGA COMPLEX (SPC) in the PAGA HAT complex [28] indicating that MtING functions extend beyond NuA4. This is supported by the fact that there were many more up regulated DEGs than down regulated in *Mting2-2* and *Mting1-7 Mting2-2* mutants compared to WT (Table S2, Fig. 4B). This indicates that the MtINGs overall act as repressors of gene expression rather than activators [27].

### Analysis of the *Mting1-1Mting2-11* double PHD mutant indicates that the PHD fingers do not contribute strongly to MtING function in flowering and development

Our previous work with *Mting2* PHD finger single mutants indicated that, unexpectedly, the conserved PHD finger was not crucial for its function, because these single mutants grew and flowered similarly to WT [27]. To test if the loss of the MtING2 PHD finger was being compensated for by MtING1, we made the *Mting1-1 Mting2-11* PHD double mutant. Apart from a strong loss of trichomes, the double mutant showed only a mild delay to flowering time and weak effects on development and gene expression. Therefore, the MtING PHD fingers do not appear to contribute strongly to the biological function of the MtING proteins in *Medicago* in an otherwise WT background.

## Conclusions

Our work indicates that *MtING1* and *MtING2* have overlapping and complementary functions with a combined essential role in the transition to flowering in floral inductive VLD conditions and in growth and development. However, interestingly, despite their conservation, the MtING PHD double mutant plants have much milder phenotypes than the double knockout plants. This implies that the PHD fingers of the MtINGs are not crucial for MtING function in flowering and growth, in an otherwise WT plant. These results raise the possibility that other Medicago PHD finger proteins may compensate in their absence. These results also implicate the other conserved domain, the N-terminal ING domain, as perhaps the most important element for MtING function. Future work to examine the overlapping and complementary roles of the MtINGs should investigate whether the proteins are components of chromatin modifying complexes such as *Medicago* NuA4 and/or PAGA HAT complexes and what genes might be direct targets of these complexes, providing valuable insights into the fundamentals of epigenetic regulation in plants. More broadly, based on the foundations provided by our work, it will be important to expand investigation of the functions of the *ING* genes in growth, development and flowering in other plants. For example, recently, other NuA4 associated genes have been shown to affect key agronomic traits such as fertility and flowering time in both *Oryza sativa* L. (rice) and *Zea mays* L. (maize) [56–58] indicating that more research on the *ING* genes in key crop species might provide a strong foundation for improving crop varieties to meet future agricultural challenges.

## Supplementary Information

Supplementary Material 1: Table 1. List of primers used

Supplementary Material 2: Table 2. Differentially expressed genes in the apex and leaf of all Mting mutants compared to WT R108, selected based on a cut-off of log2fold-change ≥ 1 or ≤ -1 and adjusted *p*-value ≤ 0.05.

Supplementary Material 3: Table 3. Gene ontology analysis of differentially expressed genes in all Mting mutants. Top 30 pathways from GO categories Biological Process are presented

Supplementary Material 4: Table 4. Expression of candidate differentially expressed genes identified from the apex and leaf of all Mting mutants in RNA-seq (a), the Cheng list [13] (b), and candidates validated by RT-qPCR (c).

Supplementary Material 5: Table 5. Expression of Medicago homologs of Arabidopsis genes targeted by NuA4 from Bieluszewski et al . [32].

## Data Availability

The datasets generated during the current study are available in the GEO repository (GSE277907).
